# Analysis of *Ochetobius elongatus* (Kner) Dietary Habits Based on Digestive System Morphology, Histology, and Intestinal Content Sequencing Technology

**DOI:** 10.3390/ani16091369

**Published:** 2026-04-29

**Authors:** Feng Gao, Zhiliang Zuo, Qifan Wu, Hewei Xiao, Zhitao Peng, Li Zou, Guomin Jiang, Xing Tian, Zhifeng Feng, Xuan Xie, Lu Tian

**Affiliations:** 1 Hunan Fisheries Research Institute and Aquatic Products Seed Stock Station, Changsha 410005, China; gfeng0511@163.com (F.G.); zuozliang@163.com (Z.Z.); wuqifvan@hunaas.cn (Q.W.); x42643934@163.com (H.X.); pengztao@126.com (Z.P.); lily_zou@126.com (L.Z.); smdeng1126@hunaas.cn (G.J.); tianx2323@163.com (X.T.); fzf1987@hunaas.cn (Z.F.); 2 Yuelushan Laboratory, Hunan Academy of Agricultural Sciences, Changsha 410153, China

**Keywords:** *Ochetobius elongatus*, morphology, histology, eDNA meta-barcoding, metagenomics, diet

## Abstract

*Ochetobius elongatus* (Kner) is a migratory fish found in the Yangtze River basin and areas south of it, and listed as a critically endangered (CR) fish on the China Red List of Vertebrates. To achieve group recovery and artificial breeding, this study investigated the dietary characteristics of *O. elongatus* based on high-throughput sequencing of its intestinal contents, and its digestive system morphology, and its histology. Results showed that the digestive system of *O. elongatus* lacked a stomach and mainly consisted of the oropharynx, pharyngeal teeth, esophagus, intestine, and anus. The gut index was 0.88, with clear segmentation of the foregut, midgut, and hindgut, and the visceral mass index was 7.35%. Histological analysis of the digestive system revealed the presence of keratinized dental plates or pharyngeal teeth in the pharynx, as well as a high density of taste bud cells in the soft palate of the oral cavity. The surface layer of the intestinal villi contained numerous mucous cells, with the average number of mucous cells per villus gradually increasing from the esophagus to the hindgut, and the foregut having the longest and most abundant mucosal folds. The esophagus exhibited well-developed circular and longitudinal muscle layers, while in the hindgut, both the circular and longitudinal muscle layers were slightly thicker than those in the midgut. High-throughput sequencing of the intestinal contents of *O. elongatus* revealed the following phyla based on 18S V4 meta-barcoding: Chlorophyta, Diatoms, Arthropoda, Basidiomycetes, and Ascomycetes, with the genus *Hypophthalmichthys* and algae being the main classifications. In contrast, based on COI meta-barcoding, the study newly identified the phyla Cnidaria and Mollusca, with the genera *Chlorophyta*, *Scenedesmus*, *Pectinodesmus*, and zooplankton such as *Pseudodiaptomus*. Metagenomic sequencing revealed that the gut microbiota at the phylum level was predominantly composed of Pseudomonadota, Ascomycota, Basidiomycota, Chytridiomycota, and Bacillota, with key genera including *Cetobacter*, *Pseudomonas*, *Acinetobacter*, *Aeromonas*, and *Clostridium*. This study indicates that *O. elongatus* is an omnivore with carnivorous tendencies. Basic biological research on *O. elongatus* is of great significance for the restoration of the population, artificial breeding, and the development of its artificially formulated feed. It also provides important data for the formulation of biodiversity conservation measures.

## Simple Summary

*Ochetobius elongatus* was once an important commercial fish species in the Yangtze River basin. It subsequently became an endangered species, but has reappeared in reports in recent years. Currently, research on *O. elongatus* focuses primarily on genetic diversity, whilst studies on its basic biology—such as analyses of its feeding habits—are scarce; such research offers important insights into the species’ current survival status. In this study, the digestive system of *O. elongatus* was dissected, relevant morphological parameters were measured, and histological samples of the digestive tract were collected. We then performed high-throughput sequencing analysis of the intestinal contents using meta-barcoding and metagenomic methods. The results indicate that *O. elongatus* possesses sharp, well-developed pharyngeal teeth, a relatively short intestine, and a low visceral mass index. The types of mucous cells and tissue structure in the foregut, midgut, and hindgut suggest a digestive system structure characteristic of an omnivore with carnivorous tendencies; high-throughput sequencing analysis of intestinal contents revealed that *O. elongatus* feeds on algae, arthropods, molluscs, and fish. These consist primarily of common biological prey such as silver carp and marsh shrimp. The microbial composition of *O. elongatus*’ gut also exhibits a certain degree of similarity to that of omnivorous and carnivorous fish, and the results also indicate that *O. elongatus* is an omnivore with carnivorous tendencies.

## 1. Introduction

Fish diets are the result of adaptation to the living environment, affecting their own growth, reproduction, and other biological community structures and roles in the ecosystem [1,2,3]. The survival status of the rare indigenous fish is closely related to their diets, as diets directly relate to their nutritional needs, energy flow, and interspecific relationships. Systematic research on the diets of the rare indigenous fish is fundamental to revealing their niche differentiation, resource utilization strategies, and group dynamics mechanisms, and also provides precise feed formulations and ecological regulation schemes for artificial domestication and breeding, habitat restoration, and stock enhancement [4]. Numerous studies on fish diets and their influencing factors have been conducted both in China and in other parts of the world [5,6,7]. In recent years, China has made significant progress in the investigation, artificial breeding, and conservation practices of the rare indigenous fish resources, but systematic research on their dietary ecology remains relatively weak, lacking in-depth research on feed selection preferences and nutritional niche differentiation. Existing research mainly focuses on group monitoring and reproductive biology, while the lack of dietary data limits the efficiency of conservation strategies and also affects the industrialization of the rare indigenous fish species.

*Ochetobius elongatus* (Kner), a critically endangered (CR) species on the China Red List of Vertebrates [8], belongs to the order Cypriniformes, family Cyprinidae, subfamily Leuciscinae, and genus *Ochetobius*, and is the only species in its genus. *O. elongatus* is characterized by a slender, elongated body. The mouth is terminal and protrusible. All fins, except the dorsal fin, exhibit a slight orange-red hue. Morphologically, it lacks both dorsal spines and an abdominal keel. It is distributed in the Yangtze River basin and areas south of it. In recent years, the *O. elongatus* has been monitored in Dongting Lake, Poyang Lake, and the Yangtze and Pearl River basins in China [9,10,11]. *O. elongatus* has also been documented in tributaries of the Dongting Lake system within Hunan province of China, including the Xiang, Zi, Yuan, and Li rivers. Due to its declining populations, it is currently listed as a key protected wildlife species in several provinces across China. The earliest report on the reproductive biology of *O. elongatus* was published in the 1970s [12]. In recent decades, the population of *O. elongatus* has shown a gradual increase; however, the collection of viable specimens remains challenging. Current research on the *O. elongatus* mainly focuses on genetic diversity analysis, with a notable lack of targeted studies on its dietary [13,14,15].

Research has found that morphological characteristics of the fish digestive system can be used as a basis for judging its diet [16,17,18]. Fish diets are closely related to gut index [19,20], and the size of the intestinal surface area of fish is correlative to differences in interspecific and intraspecific nutritional levels [21]. The histological characteristics of the fish digestive system are consistent with its diet and digestive mechanisms. Morphological characteristics of the digestive system and the number of mucous cells differ among fish at different trophic levels [22]. The presence of numerous taste bud cells in the esophagus suggests that fish can select food based on taste [23]. Studies have found that the intestinal villi branching and structure are more complex in carnivorous fish [24], and the gill rakers of filter-feeding fish have fine teeth or a comb-like structure [23]. The digestive characteristics of the fish esophagus are significantly correlated with feeding preferences and digestive function [25,26,27]. Research on the morphology and histology of the digestive system will enhance our understanding of fish feeding and digestive strategies. Studies on fish diet frequently utilize microscopic examination of gut contents and stable isotope analysis. In recent years, with the rapid development of high-throughput sequencing technology, eDNA meta-barcoding and metagenomics technologies have also been widely applied to animal dietary studies [28,29], thereby enriching the methodological toolkit for investigating fish diets. eDNA meta-barcoding technology utilizes targeted amplification of specific gene regions (e.g., 12S, 18S, COI) coupled with high-throughput sequencing, enabling highly sensitive and efficient identification of prey organisms from gut content samples [29]. In a study of the feeding habits of predatory fish in Poyang Lake, meta-barcoding technology made it possible to newly detect prey species, such as *Megalobrama amblycephala* and *Pseudobrama simoni*, revealing a more refined food web structure [30]. In the feeding analysis of juvenile *Hyporhamphus sajori*, meta-barcoding technology successfully identified multiple phyla, including Arthropoda, Chlorophyta, and Bacillariophyta, and revealed a dietary shift from omnivory to zooplankton dominance during development, underscoring the distinct advantages of this technology for studying early-life-stage feeding ecology [31]. The gut microbiota of fish varies with their feeding habits, influenced by dietary patterns and food composition [32]. Metagenomics analyzes the function and diversity of this gut microbial community, elucidating piscine nutritional strategies and digestive adaptations through the lens of food-microbe-host interactions [33]. For instance, a study on the diet of the yellowfin seabream (*Acanthopagrus latus*) in Daya Bay found its gut microbiota to be dominated by Proteobacteria, Firmicutes, and Bacteroidetes [34]. These microbial communities participate in digesting animal-derived feed and benthic algae by producing enzymes such as proteases, lipases, and chitinases, offering a new perspective for understanding the species’ feeding ecology and informing its health management. In the study of the diet of rare indigenous fish, the application of meta-barcoding and metagenomics technologies is particularly important. Due to the scarcity of species and the difficulty in sampling, traditional methods may be unsuitable for systematic analysis because it is difficult to obtain sufficient samples. With the improvement in molecular databases and the optimization of bioinformatics methods, meta-barcoding and metagenomics technologies will be widely used in the study of the diet of rare indigenous fish.

This study focuses on the wild or farmed *O. elongatus* groups in the Dongting Lake Basin. Based on high-throughput sequencing of intestinal contents combined with morphological and histological analysis of the digestive system of *O. elongatus*, this study aims to elucidate its feeding behavior and provide fundamental data for nutritional feed development. These findings lay an important foundation for the recovery of *O. elongatus* populations and their artificial breeding.

## 2. Materials and Methods

### 2.1. Samples Collection

Five individuals of the wild *O. elongatus* (25.38 ± 3.78 cm in total length) were collected during the 2024 Yangtze River Basin Aquatic Biological Resources Monitoring (Hunan Station)—a regulated survey requiring a legal fishing permit. Sampling was conducted in two interconnected habitats within the Dongting Lake system as follows: three individuals were obtained from the Lishui River estuary, which flows into South Dongting Lake, and two individuals from Wanzi lake, located within South Dongting Lake itself. All sampling sites are hydrologically connected to the Yangtze River network, which ultimately discharges into the sea. Four individuals of the farmed *O. elongatus* (37.93 ± 5.74 cm in total length) were also collected. During September and October 2024, intestinal contents and other samples were collected from all farmed or wild *O. elongatus* individuals. Following collection, the intestinal content samples were immediately flash-frozen on dry ice and stored for later use. The farmed *O. elongatus* originated from wild-caught fish fry approximately 3–4 days post-hatching, which were accidentally collected from the Xiang River in 2020 during a survey targeting the original stocks of the four major Chinese carps. These fry have since been reared continuously in an ecological co-culture system at the Hunan Provincial Aquatic Germplasm Conservation Farm. By 2024, the individuals had reached sexual maturity.

### 2.2. Morphological of the Digestive System

Prior to imaging, all individuals were anesthetized with tricaine methanesulfonate (MS-222, Sigma-Aldrich, St. Louis, MO, USA). Following this procedure, the pharyngeal teeth and other skeletal structures of *O. elongatus* (*n* = 2) and *Elopichthys bambusa* (*n* = 2) were visualized and photographed using a high-frequency X-ray machine (Shimadzu XUD150L-F, Kyoto, Japan) to facilitate comparative morphological analysis. Following dissection, the total length, body length, intestinal length, visceral mass, and body weight of *O. elongatus* were measured. The head was boiled in water for 20 min to remove the intact pharyngeal teeth. Then close-up visualization was performed to illustrate the head from multiple angles, as well as the detailed features of the pharyngeal teeth, gills, and intestinal tract. The *O. elongatus*’ (*n* = 9) intestinal length ratio and visceral weight ratio were quantified following the statistical methodology described by Ozaki [35]. The relative ratio of intestinal length to body length represented the gut index, and the relative ratio of visceral mass weight to body weight represented the visceral mass index.

### 2.3. Histological Characteristics of the Digestive System

Tissue samples of the digestive system of the farmed *O. elongatus* were collected (*n* = 4). After dissection, the lips, palate or pharynx, esophagus, foregut, midgut, and hindgut were collected. Given the short esophagus of *O. elongatus*, it was sequentially divided in an anterior-to-posterior direction into three segments (esophagus A, B, and C, respectively) to ensure comprehensive anatomical coverage and prevent the oversight of any functionally or structurally distinct regions. These tissues above were fixed in 4% paraformaldehyde for at least 24 h, then dehydrated using a Leica HistoCore PEARL automatic dehydrator (Leica Biosystems, Deer Park, IL, USA), cleared in xylene, and embedded in paraffin. Sections were prepared using a Leica RM2016 microtome (Leica Biosystems, Deer Park, IL, USA), with a section thickness of 5 μm. After manual slide preparation and drying, the slides were stained with hematoxylin-eosin (HE), alcian blue (AB, pH = 2.5), periodic acid-Schiff reagent (PAS), and AB-PAS. The slides were subsequently mounted with neutral resin and scanned using a whole-slide images (WSIs) scanner (KFBIO Co., Ltd., Ningbo, China) to produce high-resolution digital images. Images with scale bars were visualized using K-Viewer (Konfoong Bioinformation Tech Co., Ltd., Ningbo, China. version 1.0.5, 2024. https://www.kfbio.cn/k-viewer/ (accessed on 2 August 2024)). For quantitative analysis, three randomly selected tissue cross-sections from each tissue were assessed. We examined three non-consecutive tissue cross-sections from each region (esophagus, foregut, midgut, hindgut) for each individual, and these mean values (*n* = 12, per region) were subsequently used for the statistical comparisons among regions presented in the results. The distinct staining patterns observed in this study—with mucous cell types I, II, III, and IV appearing red, blue, purplish-red, and bluish-purple, respectively—were attributed to the varying proportions of neutral and acidic mucopolysaccharides present in these cell types, consistent with the classification previously described by Shi and Zhao [36]. Within each cross-section, the number of intestinal villi and the count of mucous cells on each villus were directly quantified from the digital whole-slide images opened in K-Viewer, and the average number of mucous cells per villus was calculated. Morphometric parameters, including average intestinal villus length, villus spacing, thickness of the muscular layer, and intestinal diameter, were measured using Adobe Photoshop (Adobe Inc., San Jose, CA, USA, Adobe Photoshop, 2024) by tracing straight-line distances between two defined points, with the embedded scale bars serving as the reference for calibration. To minimize measurement variability, all quantification and counting procedures were performed consistently by a single investigator. Statistical analysis was performed using SPSS 22 (IBM Corp., Armonk, NY, USA, IBM SPSS Statistics (version 22.0), 2013), with a one-way analysis of variance (ANOVA) and Tukey’s post hoc test used to evaluate significant differences (*p* < 0.05).

### 2.4. Analysis of the Diet Based on eDNA Meta-Barcoding Technology

After dissecting wild and farmed *O. elongatus*, the entire intestinal contents were placed in 2 mL cryovials and immediately stored on dry ice until DNA extraction was performed. This was followed by 18S V4 and COI fragment amplification and sequencing. For the DNA extraction and PCR amplification in the meta-barcoding technology, total DNA was extracted from the intestinal contents according to the instructions of the CretMag™ Power Soil DNA Kit (CretBiotech, CNA26190S, Suzhou, China). This method was adapted from established protocols for microbial DNA isolation from complex environmental samples [37]. The quality of DNA extraction was assessed using 1% agarose gel electrophoresis, and DNA concentration and purity were determined using a NanoDrop2000 (Thermo Fisher Scientific, Waltham, MA, USA). The amplification program by PCR instrument (Langji A200, Hangzhou, China) was as follows: 94 °C pre-denaturation for 2 min, 30 cycles (94 °C denaturation for 30 s, 55 °C annealing for 30 s, 72 °C extension for 30 s), followed by a stable extension at 72 °C for 10 min, and finally storage at 4 °C. The PCR reaction system was as follows: 25 μL of 2x ES Taq MasterMix (Dye), 2 μL each of forward and reverse primers (10 μM), 10 ng of template DNA, and ddH2O to a final volume of 50 μL. Each sample was replicated in triplicate. After mixing the PCR products from the same sample, the PCR products were recovered using a 2% agarose gel. The recovered products were purified using the AxyPrep DNA Gel Extraction Kit (Axygen, AP-GX-50, Union City, CA, USA), detected by 2% agarose gel electrophoresis, and quantified using a Quantus™ Fluorometer (Promega, E6150, Madison, WI, USA). Library construction was performed using the NEXTflex™ Rapid DNA-Seq Kit (Bioo Scientific, Nova-5188-03, Austin, TX, USA) as follows: (1) adapter ligation; (2) removal of adapter self-ligated fragments using magnetic beads; (3) enrichment of the library template using PCR amplification; (4) recovery of the PCR products using magnetic beads to obtain the final library. Sequencing was performed using the Illumina NovaSeq PE250 platform (Wuhan Benabio Co., Ltd., Wuhan, China). After data splitting and quality control, amplicon sequence variants (ASVs) characterization, species annotation, α and β diversity analysis, intergroup difference analysis, correlation analysis, and functional prediction analysis were conducted. Meta-barcoding technology was used to explore plankton, algae, and fish or shrimp components in the contents of *O. elongatus.* Universal primers: 18S V4F, 5-CCAGCASCYGCGGTAATTCC-3; 18S V4R, 5-ACTTTCGTTCTTGATYRA-3; COI F, 5-GGWACWGGWTGAACWGTWTAYCCYCC-3; COI R, 5-TANACYTCNGGRTGNCCRAARAAYCA-3. Amplifying the 18S V4 fragment mainly detects plankton and algae, while amplifying the COI fragment mainly detects zooplankton, fish, shrimp, and other organisms.

### 2.5. Analysis of the Diet Based on Metagenomic Technology

Sample collection was the same as in Section 2.4. Metagenomics was used to reveal the diversity and relative abundance of microbial species involved in the digestion and absorption of food in the gut of *O. elongatus*. DNA was isolated and purified using a magnetic bead-based fecal genomic DNA extraction kit (TIANGEN Biotech, DP302-02, Beijing, China). DNA concentration was then detected using Qubit 1X dsDNA HS Assay Kits (Invitrogen, Q33230, Carlsbad, CA, USA). 200 ng of QC-compliant DNA was transferred to a 0.6 mL low-adsorption centrifuge column (Axygen, MCT-060-LC, Hangzhou, China), and water was added to a final volume of 52 μL to fragment the DNA. The fragmented product was purified and recovered using magnetic beads from the TruSeq library preparation kit (Illumina, 20018704, San Diego, CA, USA). The quality and fragment size distribution of the DNA were assessed using the Agilent High Sensitivity DNA Kit (Agilent Technologies, 5067-4627, Santa Clara, CA, USA) on an Agilent 2100 Bioanalyzer, following the manufacturer’s instructions. DNA libraries were constructed using the TruSeq Nano DNA LT Library Preparation Kit (Illumina, FC-121-4001, San Diego, CA, USA) following the manufacturer’s protocol. After end repair, adapter ligation, index PCR amplification, and purification, the genomic library was constructed. The DNA library was quantified on a Qubit fluorometer using the Qubit 1X dsDNA HS Assay Kit (product details as above). Finally, paired-end sequencing was performed using Illumina Novaseq XP (LC Bio Technology CO., Ltd., Hangzhou, China) according to standard operating procedures. The sequencing was performed on an Illumina NovaSeq 6000 system in paired-end 150 bp mode. The NovaSeq 6000 XP 4-Lane Kit v1.5 (Illumina, 20043131, San Diego, CA, USA) was used for lane-specific library amplification and loading, followed by sequencing using a NovaSeq 6000 S4 Reagent Kit v1.5 (300 cycles) (Illumina, 20028312, San Diego, CA, USA). Venn plots were used to compare the number of shared and endemic ASVs between wild and farmed groups. The α-diversity indices, including Chao1, observed species, Good’s coverage, Shannon, and Simpson, were calculated using QIIME 2 (version 1.9.1; http://qiime2.org/ (accessed on 4 February 2025) for each taxonomic level and visualized as rarefaction curves. Using the Wioxon rank-sum test statistical method, β-diversity was visualized by calculating Bray–Curtis distances and using principal coordinates analysis (PCoA) and nonmetric multidimensional scaling (NMDS). Analysis of similarity (ANOSIM) and permutational multivariate analysis of variance (PERMANOVA/Adonis) were then applied based on the UPGMA clustering results to evaluate statistical differences in community structure. Linear discriminant analysis effect size (LEfSe) was subsequently performed to identify differentially abundant taxa across groups, with a screening threshold of LDA > 3.0 and *p* < 0.05. Spearman correlation analysis was performed for species at each taxonomic level of microbiota by the R package ggplot2 (version 3.2.0) and ggnetwork (version 0.5.13), and relationship pairs with |rho| > 0.8 were shown by network plots.

## 3. Results

### 3.1. Morphology of the Digestive System

**Figure 1:**
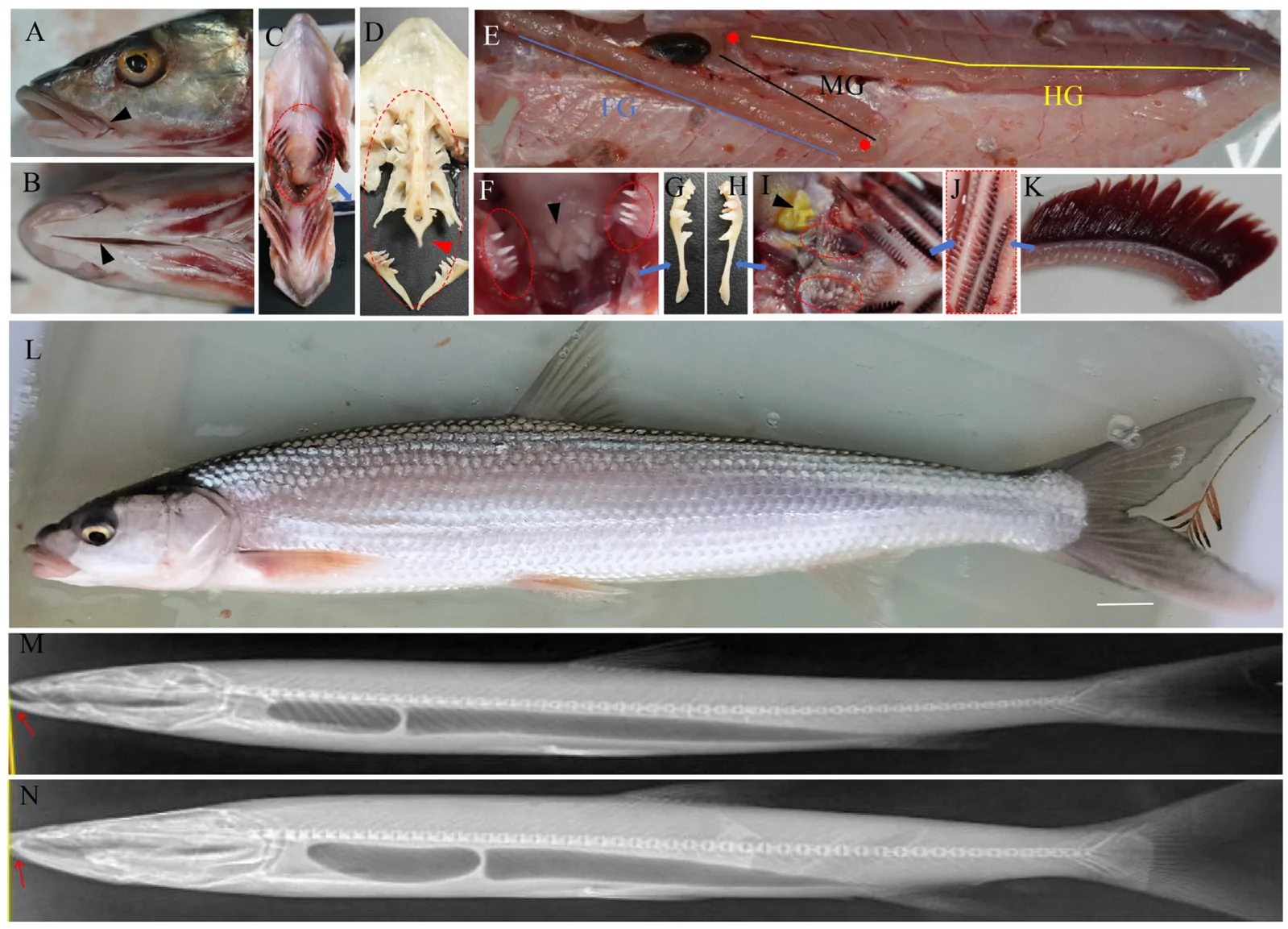
Anatomical observation of digestive system of *O. elongatus*, and radiographic comparison of *O. elongatus* and *E. bambusa*. (**A**) Rostral view. (**B**) Ventral view. (**C**) Pharyngeal cavity anatomical diagram. (**D**) Detailed anatomy of the pharyngeal jaw apparatus after removal of surrounding musculature. (**E**) Digestive tract (FG: foregut, MG: midgut, HG: hindgut), and the red solid circles indicate the points of intestinal curve. (**F**) Lateral view of pharyngeal teeth and callus pad. (**G**) Left pharyngeal tooth. (**H**) Right pharyngeal tooth. (**I**) In situ, undissected view of the complete pharyngeal teeth. (**J**) Gill rakers. (**K**) Gills. (**L**) The side view of *O.elongatus*, Scale = 2 cm. (**M**) *O. elongatus* X-ray. (**N**) *E. bambusa* X-ray. The red arrows in picture M and N: differences in internal structure of kiss tip between *O. elongatus* and *E. bambusa*.

The *O. elongatus*’ digestive system includes the digestive tract and digestive glands. The digestive tract consists of the oropharyngeal cavity, esophagus, and intestines. The relative length of the *O. elongatus*’ oropharyngeal cavity is shorter than that of the *E. bambusa*, and the *O. elongatus*’ oral fissure is shorter than that of the *E. bambusa*, ending at the anterior margin of the eye (Figure 1A). There is a narrow slit at the ventral view (Figure 1B). The pharyngeal bones of the *O. elongatus* are mainly concentrated in the upper part (Figure 1C), and the interior contains hollow, pen-tip-shaped skeletal tissue (Figure 1D). The *O. elongatus*’ intestine has two bends, as indicated by the red solid circles, arranged in an “S” shape. The foregut, midgut, and hindgut are clearly segmented, with the midgut being the shortest (Figure 1E). The digestive system lacks a stomach, but there is an enlarged foregut. Other anatomical details such as pharyngeal teeth and gills are shown in Figure 1F–K. The *O. elongatus*’ jaws are similar to that of the grass carp or common carp, with well-developed upper and lower jaws that turn outwards, and the lips and fin rays are light red (Figure 1L). The upper and lower jaws are short, with the upper jaw slightly longer than the lower jaw. A significant difference between the *O. elongatus* and the *E. bambusa* is that the latter has a sharp, tooth-like structure, as shown by the red arrows in Figure 1M,N. The relative length of the swim bladder is significantly greater in the *O. elongatus* than in the *E. bambusa*, while the anterior swim bladder chamber is shorter (Figure 1M,N). The pharyngeal cavity of the *O. elongatus* is significantly shorter in proportion to its body length than that of the *E. bambusa*. The *O. elongatus*’ tongue is underdeveloped, slightly raised, and integrated with its oral cavity. Its pharyngeal teeth are well-developed, with both sides arranged symmetrically. Each side has four rows of sharp, canine-like structures, with the front row having much longer and pointier teeth. Some pharyngeal teeth are movable and well fitted into the oral muscles. The esophagus is relatively short, with a pharyngeal bulb located at the anterior end and directly above the space between the left and right pharyngeal teeth. This pharyngeal bulb is covered with a keratinized callosity pad and has numerous tooth-like projections of varying sizes (Figure 1F). The pharyngeal teeth are in close contact with the callosity pad. The intestine of the *O. elongatus* is relatively short, with a gut index of 0.88 ± 0.05 (*n* = 4) and a relatively low visceral mass index of 7.35 ± 0.20% (*n* = 4).

### 3.2. Histological Characteristic of the Digestive System

**Figure 2:**
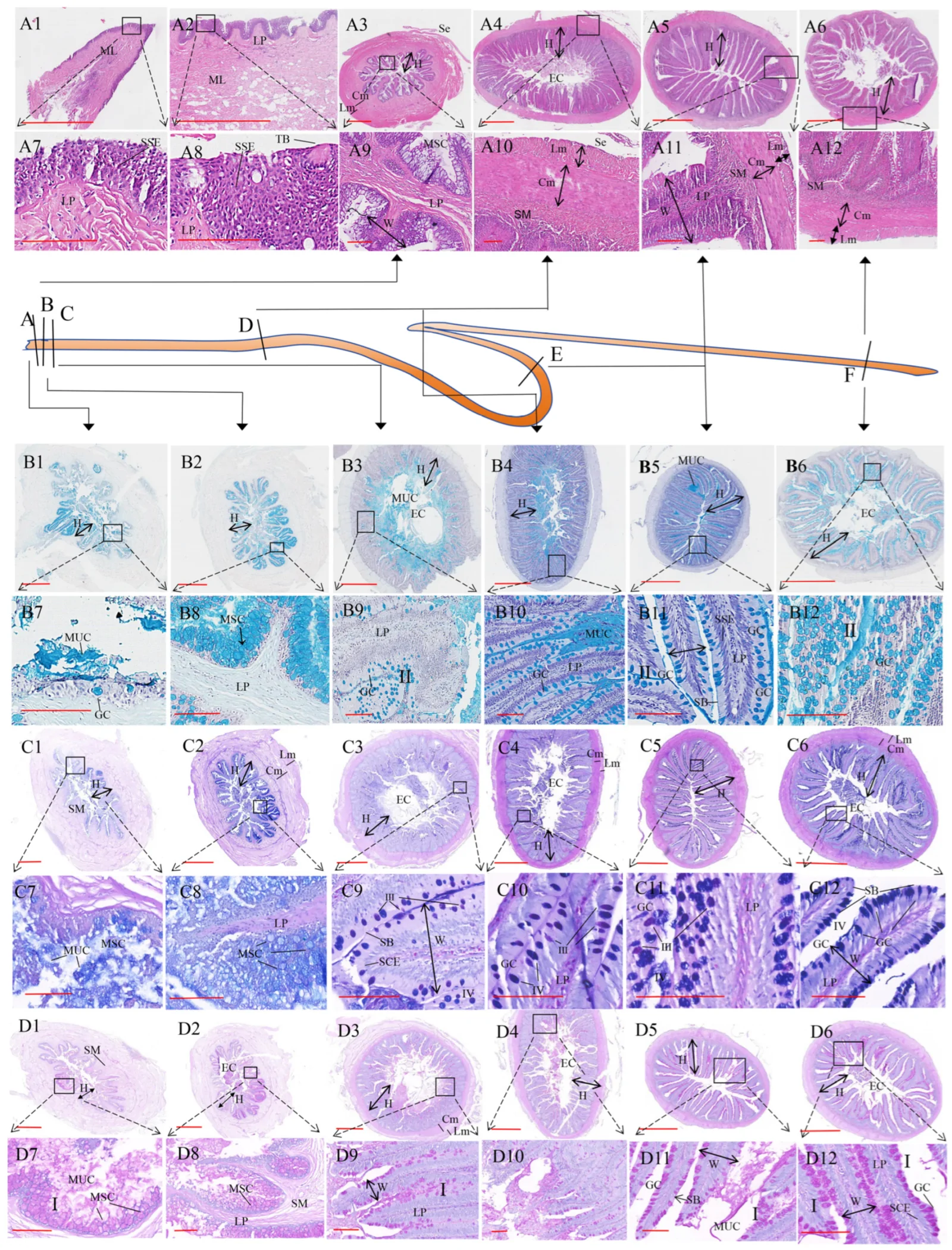
Analysis of hematoxylin-eosin (HE), alcian blue (AB), periodic acid-Schiff reagent (PAS), and AB-PAS staining of tissue sections of the digestive system of the *O. elongatus*. (**A1**–**A12**): hematoxylin-eosin (HE) staining. (**B1**–**B12**): alcian blue (AB, pH = 2.5) staining. (**C1**–**C12**): AB-PAS combined staining. (**D1**–**D12**): PAS-periodic acid-Schiff staining. (**A1**) Lip. (**A2**) Upper pharynx. (**A3**) Esophagus B. (**A4**) Foregut. (**A5**) Midgut. (**A6**) Hindgut. Low magnification → High magnification: A1→A7, A2→A8, A3→A9, A4→A10, A5→A11, A6→A12, and similarly B1–B12, C1–C12, D1–D12. Esophagus A: B1, B7, C1, C7, D1, D7. Esophagus B: A3, A9, B2, B8, C2, C8, D2, D8. Esophagus C: B3, B9, C3, C9, D3, D9. Foregut: B4, B10, C4, C10, D4, D10. Midgut: B5, B11, C5, C11, D5, D11. Hindgut: B6, B12, C6, C12, D6, D12. Scale A1–A6, B1–B6, C1–C6, D1–D6: 1000 um. Scale A8–A12, B8–B12, C8–C12, D8–D12: 100 um. A,B,C,D,E,F: Points shown at each cross-section position of the sample. H: Villus height. W: Villus width. Cm: Circular muscle. Lm: Longitudinal muscle. EC: Esophageal chyme. SB: Striate border. GC: Goblet cells. ML: Muscular layer. SM: Submucosa. LP: Lamina propria. SSE: Stratified squamous epithelium. Se: Serosa. MSC: Mucous cells. MUC: Mucous secretions.

The oropharyngeal cavity of the *O. elongatus* begins at the lips and extends to the esophagus (Figure 1C). The epithelium of the oropharyngeal cavity is a continuation of the epidermis, and from the surface to the interior, it contains stratified squamous epithelium, squamous epithelial cells, lamina propria, submucosa, and muscular layer (Figure 2(A1,A2,A7,A8)). Taste buds are also distributed on the surface of the oropharyngeal cavity (Figure 2(A8)), with more in the pharyngeal wall near the pharyngeal teeth. The lamina propria is rich in immune cells (Figure 2(C9)), and the mucosa has many villous folds, which are intestinal villi formed by the inward folding of the mucosa, and are branched or finger-like (Figure 2(A3,A4,A9,A10)). The surface layer of villi contain numerous goblet cells or mucous cells (Figure 2(B7–B12,C7–C12,D7–D12)). The muscular layer contains the circular muscle and longitudinal muscle (Figure 2(A10,A11,A12,C6)). The esophagus possesses a well-developed muscular layer, with both circular and longitudinal muscles being thickest in this region (Table 1). The esophagus is characterized by a notably thicker circular muscle layer compared to its longitudinal muscle, and possesses the thickest lamina propria along the entire digestive tract (Figure 2(B8,B9), Table 1). In the hindgut, both the circular and longitudinal muscle layers were slightly thicker than those in the midgut. The length of the intestinal villi shows no significant difference among the foregut, midgut, hindgut, esophagus A, and esophagus C. However, when normalized relative to the intestinal radius, the villi in the hindgut and midgut were significantly longer (Table 1). The number of intestinal villi increases and then decreases from the foregut to the hindgut, with the fewest villi in the esophagus A (Figure 2(B1,B7,C1,C7,D1,D7)). The villus was widest in the anterior esophagus (esophagus A) and progressively narrowed towards the hindgut, with the foregut, midgut, and hindgut all exhibiting a uniformly width narrow compared to the esophagus (Table 1, Figure 2(D1–D6)). HE staining results show the presence of taste bud cells in both the oropharynx, but none in the esophagus C to the hindgut. The foregut and hindgut clearly contain contents, while the midgut is almost entirely jejunum, and has the highest villi height relative to the intestinal radius (Figure 2(B5,C5,D5)). PAS and AB staining revealed mucous cells of types I and II, respectively. Numerous positive cells were observed from the esophagus to the hindgut, with a decrease followed by an increase, and the hindgut showing the highest number of positive cells. Interestingly, mucous cell type I showed no significant difference in distribution across the entire digestive tract (Table 1). Combining the two staining methods, AB-PAS staining, revealed a gradual increase in mucous cells II and IV in the foregut, midgut, and hindgut, with type IV mucous cells significantly more numerous than type III. The proximal esophagus lacked type III mucous cells, a feature compensated for by an abundance of free staining mucus (Figure 2(C7,C8), Table 1).

### 3.3. The Dietary Composition of Intestinal Contents Based on eDNA Meta-Barcoding

**Figure 3:**
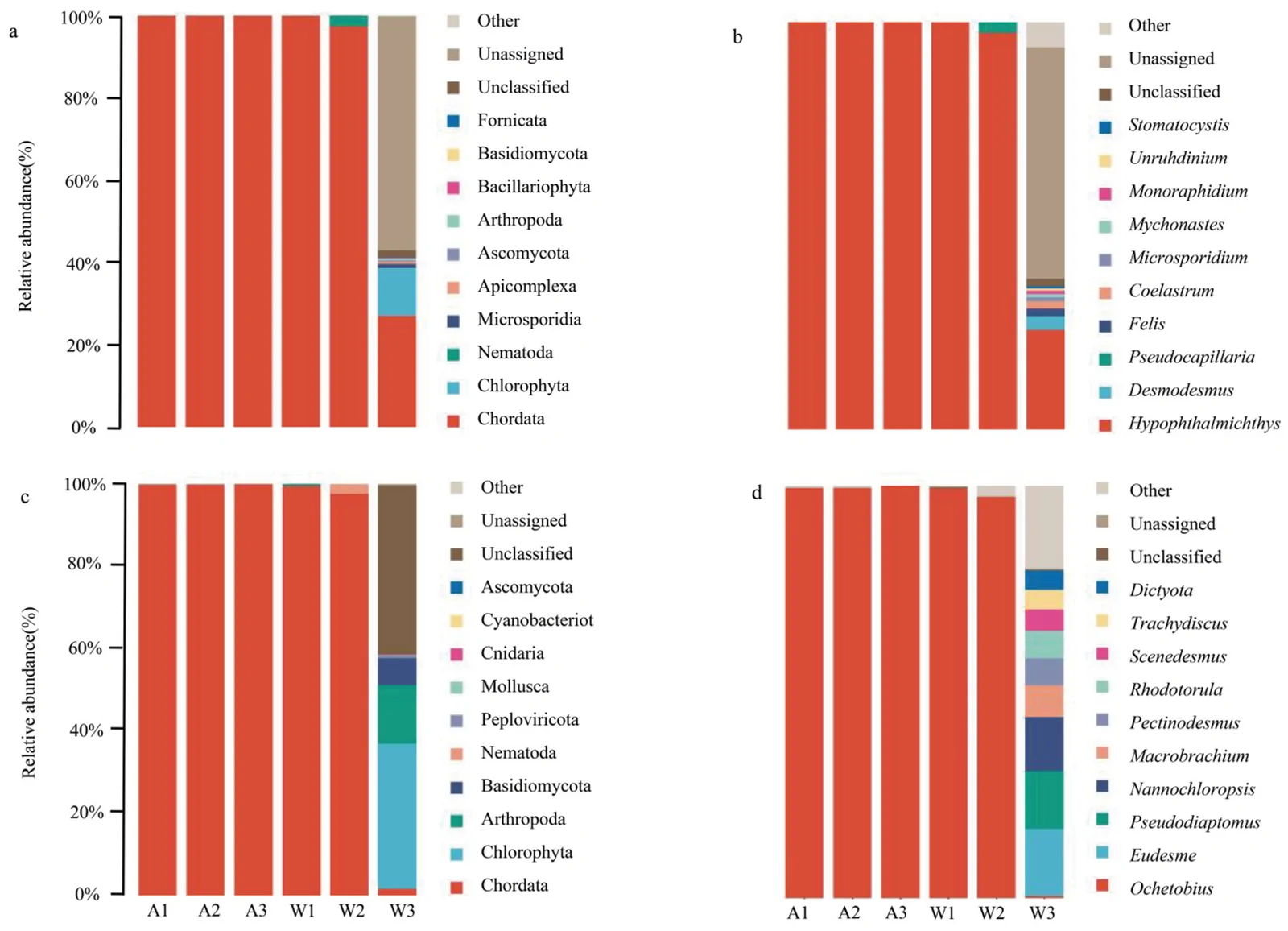
The annotation and relative abundance of phylum and genus level classification of *O. elongatus* intestinal contents based on 18S V4 and COI meta-barcoding amplification sequencing. (**a**) Phylum level of 18S V4. (**b**) Genus level of 18S V4. (**c**) Phylum level of COI. (**d**) Genus level of COI. A1–A3: farmed groups. W1–W3: wild groups.

18S V4 was used as a molecular marker to analyze the planktonic and algal composition of the intestinal contents of wild and farmed *O. elongatus*. After sequencing all samples, barcode identification yielded a total of 597,158 paired reads, with each sample generating over 96,754 paired reads, averaging 99,526 reads. The Q20 and Q30 values exceeded 98% and 94%, respectively, indicating high sequencing quality. Venn diagrams were used to compare shared and unique ASVs between the wild and farmed groups. The results showed that only four shared characteristics existed between the two groups, while the number of unique ASVs in the wild group (205 species) was significantly higher than that in the farmed group (10 species). Species annotation was performed using the corresponding reference database for each ASV, revealing that the species diversity in the wild group was significantly higher than that in the farmed group. The COI gene was also used as a molecular marker to analyze the composition of the intestinal contents, revealing the presence of bacteria, algae, arthropods, coelenterates, mollusks, and vertebrates. The results showed that both groups shared four species, but the number of unique species was significantly higher in the wild group (216 species) than in the farmed group (26 species). α-diversity was also significantly higher in the wild group than in the farmed group. Sequencing of two meta-barcode fragments revealed that at the phylum and genus levels, the top 10 phyla by relative abundance mainly belonged to Mollusca, Vertebrata, Chlorella, Protozoa, and Arthropoda, including genera such as *Hypophthalmichthys*, *Ochetobius*, *Desmodesmus*, *Eudesme*, and *Pseudocapillaria.* The 18S V4 fragment analysis showed that *Hypophthalmichthys* was the dominant genera, accounting for over 99% (Figure 3a,b). The COI fragment analysis revealed that *Ochetobius*, *Macrobrachium*, and various algal were the main genera (Figure 3c,d).

### 3.4. Metagenomic Sequencing

**Figure 4:**
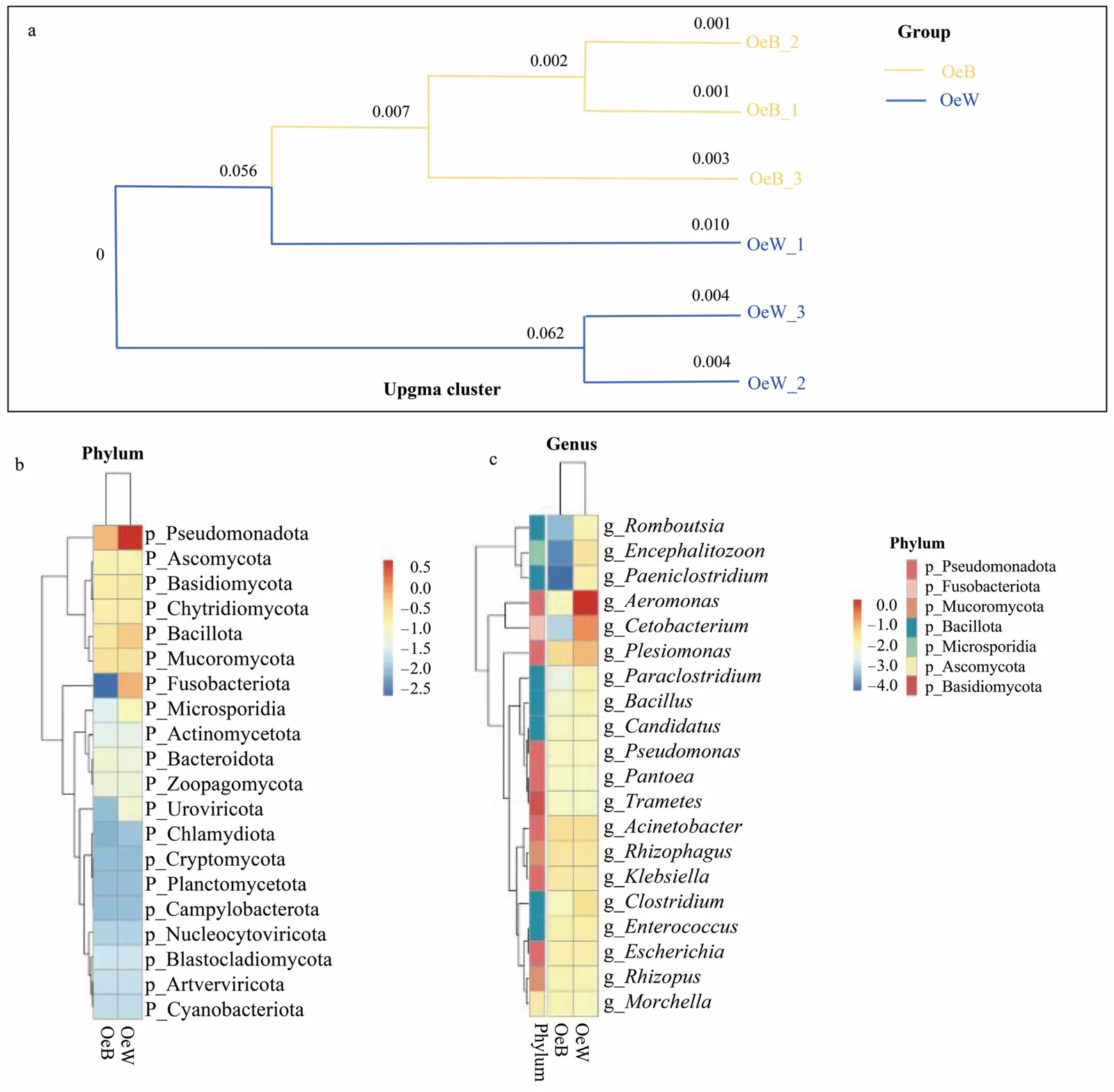
The cluster analysis and biodiversity composition the of the gut microbiota of the *O. elongatus* based on metagenomic sequencing. (**a**) Cluster analysis. (**b**) Phylum level annotation. (**c**) Genus level annotation. OeB: farmed group. OeW: wild group.

Metagenomic sequencing was performed on three intestinal contents samples from wild and farmed *O. elongatus* respectively, with an average of 6.59G of data per sample and an average N50 of 2411. Each dataset was assembled to obtain contigs, with contigs longer than 500 bp retained for subsequent cluster analysis by MEGAHIT software (version 1.2.9). CDS prediction was performed on the assembled contigs, which were then aligned to each unigene sequence. The relative abundance and species of each unigene group were calculated. The results showed that the number of shared unigenes between the two groups was 724,413, while the number of unique unigenes was significantly higher in the wild group (64,653) than in the farmed group (8354). The unigene with the best alignment result was selected as the species classification. α-diversity analysis was performed using Chao1, Observed_species, Goods_coverage, Shannon, and Simpson. Goods_coverage was 1 in both the wild and farmed groups. The farmed and wild groups were clearly clustered separately (Figure 4a). Both wild and farmed groups shared the same core microbial taxa, encompassing the phyla Pseudomonadota, Ascomycota, Basidiomycota, Chytridiomycota, and Bacillota, with key genera including *Cetobacter*, *Pseudomonas*, *Acinetobacter*, *Aeromonas*, and *Clostridium* (Figure 4b,c). The abundance of many *Bacillota* genera was comparable between the two groups. Functional gene annotation based on the KEGG database showed that at KEGG Level 1, the metabolic pathway was the most abundant in two groups, followed by the environmental information processing pathway. At KEGG Level 2, the pathways were mainly enriched in global and overview maps, signal transduction, carbohydrate metabolism, and amino acid metabolism.

## 4. Discussion

*Ochetobius elongatus*, along with *E. bambusa* and *Luciobrama macrocephalus*, is one of the three dominant apex predators in the Yangtze River, sharing the characteristic of an elongated, streamlined body. However, the current status of *O. elongatus* resources is under considerable pressure, prompting our investigation into its dietary ecology. Anatomical examination of *O. elongatus* showed that a distinct stomach is absent; instead, the digestive tract is characterized by a relatively enlarged esophagus and foregut, which is closely linked to food digestion and represents an evolutionary outcome within the Cyprinidae. According to the classification criteria for different diets of fish [38], the gut index of *O. elongatus* is significantly less than 1, suggesting that it is a carnivorous fish. According to the research findings on *Gobiocypris rarus* [39], *O. elongatus* was a kind of omnivorous fish. Based on above, we preliminarily determined that the *O. elongatus* was an omnivorous fish with a carnivorous tendency. According to the intestinal division method for *Pseudorasbora elongate* [40], the *O. elongatus*’ intestine displays two intestinal curves, dividing into the foregut, midgut, and hindgut, with the midgut being the shortest. Both the *O. elongatus* and the *E. bambusathe* belong to the Leuciscinae subfamily, but the *E. bambusathe* has a significantly larger maximum weight than the *O. elongatus*, which may be a result of nutritional niche differentiation [41]. X-ray results show that the *E. bambusathe* has a tooth-like sharp structures near the tip of the jaws in its oropharyngeal cavity, which may help it control large prey. The *O. elongatus* may rely more on pharyngeal teeth to control its food, and its mouth and oropharyngeal cavity are relatively shorter. Except for a few slender gill rakers on the first gill arch, the gill rakers on other gill arches are short and flat, which may explain why the *O. elongatus* rarely captures large prey. The well-developed upper and lower jaws may be related to the *O. elongatus*’ preference for inhabiting muddy lakes and feeding on bottom-dwelling shrimp, invertebrates, and insects, which may be a result of niche evolution within the order Cypriniformes [42]. Some the pharyngeal teeth of the *O. elongatus* are movable and embedded seemingly in the muscle, which may help it cope with the digestive constraints posed by the hard shells of crustacean prey, or rejecting indigestible exoskeletons. The visceral mass of *O. elongatus* is characterized by a low specific gravity, and its anatomical features are consistent with those typical of carnivorous fish [43].

The striated border structure of the fish intestine increases the surface area for digestion and absorption, facilitating food breakdown and nutrient absorption [44]. This histological study of *O. elongatus* revealed the following distinct structural adaptations: the foregut, midgut, and hindgut are characterized by well-developed striated borders, which function as the main sites for digestion and absorption. In contrast, the esophagus of *O. elongatus* lacks a striated border. This morphological feature is consistent with reports in omnivorous *Schizothorax eurystomus* [45]. Instead, the esophageal lumen is lined with free mucous secretions that lubricate the bolus, a transport function further supported by well-developed circular and longitudinal muscle layers. The digestive tract of *O. elongatus* displays the typical four-layer architecture (mucosa, submucosa, muscularis, and serosa), with the thickness and histological features of each segment varying in accordance with their specific digestive roles. This structural differentiation underlies its specialized digestive physiology. Furthermore, studies in cyprinids indicate that the development of intestinal mucous cells is closely tied to the degree of dietary specialization, supporting the link between gut morphology and feeding ecology observed here [46]. Along the digestive tract of *O. elongatus*, mucous cells were widely distributed, while the muscular layer thickness showed segment-specific variation, adaptations that likely contribute to a coordinated and efficient digestive process. For example, the proximal esophagus of *O. elongatus*, near the oropharynx, possesses well-developed circular muscles. This morphological adaptation likely serves as a mechanism to prevent reflux and ensure unidirectional transport of the food bolus to the intestinal tract, thereby facilitating its enzymatic digestion and subsequent absorption by the villi. The increased thickness of both the circular and longitudinal muscles in the hindgut muscularis may enhance intestinal peristalsis, facilitates content excretion, and strengthens the barrier between the intestinal lumen and the external environment, thereby contributing to the maintenance of internal homeostasis. Some studies have found that, compared to the long intestines of herbivorous fish, the hindgut of carnivorous fish with short intestines also plays a role in absorbing and utilizing water, electrolytes, and amino acids, thereby mitigating the constraint of a shorter intestinal tract through enhanced morphological and functional specialization [24,47]. The *O. elongatus*’ hindgut also has many different types of mucous cells, which also play a role in nutrient absorption, consistent with the structure of predominantly carnivorous fish. The midgut of *O. elongatus* is the shortest. In carnivorous fish, despite the short midgut, it serves as a critical segment for protein absorption [47]. The esophageal mucosal folds (intestinal villi) of the *O. elongatus* exhibit numerous branching structures, and the submucosa is well developed, with wide intervillous spaces, potentially effectively dividing the food bolus. This ensures sufficient contact between the esophageal mucosa and the contents for absorption, compensating for the shorter intestines of carnivorous fish and promoting optimal intestinal control over food. The *O. elongatus*’ swallowing or rejection of food also depends on taste bud cells in the oropharynx and esophagus, which is crucial for its foraging in the muddy bottom. The type of intestinal mucous cells in fish may be related to food and its digestive mechanisms [48,49]. Histological differences in mucous cells and other components in different intestinal segments of the *O. elongatus* reveal the intestinal response to the absorption of nutrients such as protein and fat. Similar types of mucous cells exist in the digestive tract of all fish, possessing functions such as lubrication, enzyme secretion, antibacterial activity, and osmotic regulation; their number is influenced by the food source. For example, the intestinal mucous cells of juvenile *Silurus meridionalis* exhibit a clear feeding response, and different segments of the intestine can promptly regulate the number of mucous cells when nutritional stress occurs [50]. The distribution density of mucous cells in different intestinal locations of *Rhabdosargus sarba* is correlated with the digestive function of the corresponding location [51]. In the digestive tract of *Cynoglossus semilaevis*, type III mucous cells are likely the main secretory cells of digestive enzymes, while type II and IV mucous cells are related to immune defense capabilities [52]. Studies have found that an increase in the number of type II mucous cells is a result of starvation stress [53]. Differences in intestinal mucous cells were observed in carp fed earthworms and duckweed, with more mucous cells in the duckweed-only group than in the earthworm-only group, and a lack of type I mucous cells in the foregut of the earthworm-only group [54]. Studies found that the number of intestinal mucous cells generally decreases sequentially from herbivorous to omnivorous to carnivorous fish [55]. In this study, the gut of the *O. elongatus* contained a high number of mucous cells, which differs from that of purely carnivorous fish. *O. elongatus* may also ingest detritus such as algae, requiring more mucous cells to lubricate their intestines. The esophagus of the *O. elongatus* contained fewer type I and II mucous cells but more free mucus, similar to the findings in carp [54], suggesting that *O. elongatus* may be feeding on invertebrates such as earthworms.

This study is the first to reveal the diet composition of the *O. elongatus* using meta-barcoding techniques from the 18S V4 region and the COI gene. Meta-barcoding technology has also been widely used in fish diet analysis in recent years [56,57]. In this study, the 18S V4 region primarily targeted plankton and algae, whereas the COI region was focused on detecting fish, shrimp, and other animal prey. Both analytical methods consistently demonstrated that the primary diet of *O. elongatus* consisted of silver carp and freshwater shrimp, namely the *macrobrachium*, with mollusks and coelenterates also being ingested as secondary prey. This result corresponds to the *O. elongatus* preference for bottom habitat and its well-developed upper and lower jaws, and is consistent with its spawning and reproduction in the upper reaches or rapids of rivers and its feeding and fattening in connected lakes. This is also consistent with the fact that both *Hypophthalmichthys* and *macrobrachiumare* present in the environments inhabited by both wild and farmed *O. elongatus*. Because our team’s preliminary research focused on the domestication and breeding of the *O. elongatus*, first-hand data on its nutrition and feed were lacking. No artificial formulated feed was administered, and the gut contents of both farmed and wild groups were relatively low. In this study, due to the limited contents, a large amount of intestinal mucosal tissue was collected, revealing substantial information about the host (*Ochetobius*). However, fewer types of dietary habits were detected, which may be related to the low feeding intensity, short intestines that empty easily, and insufficient intestinal contents. Therefore, in future research, it is necessary to use relevant software to filter out host genes to mitigate background noise and improve accuracy. We still found significantly higher α-diversity in the gut microbiota of the wild group than the farmed group, possibly due to the wider range of activity or higher opportunities for food access in the wild [58,59]. This study reveals substantial dietary overlap between farmed and wild *O. elongatus*, supporting the species’ potential for captive breeding while also indicating food-resource constraints in the wild possibly. The restricted diet of wild individuals may reflect reduced prey abundance linked to habitat disturbance, consistent with the population declines that have led to its regionally protected status. Algal traces in gut contents were likely ingested incidentally through consumption of filter-feeding prey. Although stable-isotope analysis of intestinal contents is frequently used in dietary studies [57], it proved unsuitable here due to the scarcity of identifiable gut contents and their advanced digestion. Future work with sufficient samples should incorporate eDNA meta-barcoding to obtain more precise and comprehensive dietary profiles.

Metagenomic sequencing of intestinal contents from *O. elongatus* revealed distinct gut microbiota profiles between wild and farmed groups, reflecting adaptations to their respective dietary and environmental conditions based on functional gene annotation by the KEGG of two levels. Studies indicate that gut microbiota in wild fish are often linked to lipid metabolism and unsaturated fatty acid synthesis [60], whereas in farmed fish they are associated with terpenoid and polyketide metabolism, which may underlie observed differences in muscle quality between groups [61,62,63]. Some studies have found that dietary structure affects these differences in gut microbiota composition [32,33,34]. Importantly, both wild and farmed *O. elongatus* shared several core microbial taxa common to carnivorous fishes—such as *Pseudomonas*, *Aeromonas*, *Cetobacterium*, and *Fusobacterium*—highlighting a conserved gut microbiome adapted to high-protein diets [64]. There were *Cetobacterium* spp. in the gut contents of *O. elongatus*, similar to wild *Onychostoma macrolepis* whose diet is mainly animal-based. *Cetobacterium* spp. are associated with high protein and high lipid synthesis and metabolism [65]. Studies have found that starvation stress can significantly alter gut microbiota diversity in fish [66]. Notably, a high proportion of unclassified microbiota suggests untapped functional potential within the gut microbiome of *O. elongatus*. Although α-diversity was lower in the farmed group—likely due to ecological lakes with limited prey diversity—both groups harbored overlapping microbial communities, including taxa such as Romboutsia and Paeniclostridium that are also dominant in planktivorous fishes [67]. This overlap aligns with meta-barcoding results indicating that *O. elongatus* consumes plankton, supporting its flexible feeding strategy, a characteristic of omnivorous fish. The greater within-group variation observed in wild fish probably reflects the complexity and heterogeneity of natural habitats. Collectively, these findings offer insights for the development of formulated feeds, probiotics, and domestication strategies for *O. elongatus*, a species whose gut microbiome exhibits features of both carnivorous and omnivorous fishes, underscoring its ecological adaptability and potential for sustainable aquaculture. This study may contribute to artificially domesticating and breeding *O. elongatus*, developing formulated feeds and additives, and advancing probiotic applications. Through metagenomic sequencing, we discovered that *O. elongatus* possess gut microbiota composition structures characteristic of both carnivorous and omnivorous fish.

## 5. Conclusions

Integrated morphological, histological, eDNA meta-barcoding, and metagenomic analyses of the digestive system indicate that *O. elongatus* is an omnivore fish with carnivorous tendencies. Future research should integrate electron microscopy, transcriptomics, and metabolomics to define the microstructural and molecular basis of dietary adaptation. The robustness of findings would be enhanced by cross-validating intestinal content sequencing technology with traditional methods like stable isotope analysis, while acknowledging current limitations of reference databases. This study provides a foundation for such work, which will partially inform the development of optimized feeds and breeding protocols in support of *O. elongatus* conservation.

## Figures and Tables

**Table 1:** Histological characteristics and morphometric data of the digestive tract in *O. elongatus.* Data are presented as mean values. Measurement units: μm for length; n for count. And means with different letter on the same line are statistically different (mean ± SEM).

Variable	Esophagus A	Esophagus B	Esophagus C	Foregut	Midgut	Hindgut
villus length ^1^/um	638 ± 110 b	508 ± 126 a	771 ± 140 b	773 ± 135 b	752 ± 165 b	753 ± 152 b
villi ^1^/n	20 ± 2 a	28 ± 5 b	57 ± 2 d	65 ± 9 d	34 ± 5 bc	37 ± 2 c
villus width ^1^/um	488 ± 211 c	227 ± 74 b	96 ± 17 a	43 ± 16 a	76 ± 26 a	89 ± 19 a
circular muscle thickness ^1^/um	550 ± 193 b	506 ± 223 b	170 ± 41 a	137 ± 28 a	101 ± 25 a	122 ± 32 a
longitudinal muscle thickness ^1^/um	409 ± 128 b	349 ± 47 b	446 ± 220 b	121 ± 15 a	73 ± 17 a	81 ± 10 a
intestinal radius ^1^/um	2604 ± 513 b	2532 ± 440 b	2390 ± 169 b	2374 ± 330 b	1495 ± 156 a	1594 ± 115 a
mucous cells I ^2^/n	37 ± 30	44 ± 39	25 ± 8	26 ± 7	52 ± 14	44 ± 15
mucous cells II ^2^/n	38 ± 20 ab	26 ± 11 bc	16 ± 3 a	33 ± 10 ab	37 ± 17 ab	63 ± 12 c
mucous cells III ^2^/n	0 ± 0 c	0 ± 0 c	18 ± 6 a	11 ± 3 ab	8 ± 3 b	17 ± 12 a
mucous cells IV ^2^/n	26 ± 4 a	15 ± 9 a	26 ± 8 a	15 ± 6 a	45 ± 15 b	56 ± 11 b

^1^ Values represent the average of corresponding variables per cross-section. ^2^ Values represent the average number per villus in cross-section.

## Data Availability

The data that support the findings of this study are available from the corresponding author upon reasonable request.
